# A Eficiência Ventilatória Seria a Chave para Revelar o Potencial Máximo do Teste Cardiopulmonar de Exercício?

**DOI:** 10.36660/abc.20240184

**Published:** 2024-05-17

**Affiliations:** 1 Grupo de Pesquisa em Cardiologia do Exercício Hospital de Clínicas de Porto Alegre Porto Alegre RS Brasil Grupo de Pesquisa em Cardiologia do Exercício (CardioEx) - Hospital de Clínicas de Porto Alegre, Porto Alegre, RS – Brasil; 2 LPH-Laboratório de Performance Humana Rio de Janeiro RJ Brasil LPH-Laboratório de Performance Humana, Rio de Janeiro, RJ – Brasil; 3 Universidade do Estado do Rio de Janeiro Rio de Janeiro RJ Brasil Universidade do Estado do Rio de Janeiro, Rio de Janeiro, RJ – Brasil; 4 Universidade Federal do Rio Grande do Sul Faculdade de Medicina Porto Alegre RS Brasil Programa de Pós-Graduação em Cardiologia e Ciências Cardiovasculares - Faculdade de Medicina, Universidade Federal do Rio Grande do Sul, Porto Alegre, RS – Brasil; 5 Universidade Federal do Rio Grande do Sul Departamento de Clínica Médica Porto Alegre RS Brasil Universidade Federal do Rio Grande do Sul - Departamento de Clínica Médica, Porto Alegre, RS – Brasil

**Keywords:** Ergoespirometria, Troca Gasosa, Marcadores

Se o Professor Jorge Pinto Ribeiro estivesse vivo, sem dúvida seria quem que estaria escrevendo esse minieditorial em vez de nós. Infelizmente, nós o perdemos em 2013, mas sua memória, e palavras e artigos inspiradores continuam presentes. O Professor Ribeiro era um defensor obstinado do Teste Cardiopulmonar de Exercício (TCPE). Ele escreveu vários artigos sobre o tema, incluindo um com título instigante: "*Beyond Peak Oxygen Uptake: New Prognostic Markers from Gas Exchange Exercise Tests in Chronic Heart Failure*".^1^ É uma honra mencionar que o Professor Chiappa e o Professor Stein tiveram a sorte de colaborar com ele nesse artigo.

O que aconteceu há 18 anos^1^ está diretamente relacionado ao que houve em seguida: apesar da riqueza de dados valiosos disponíveis, o TCPE continua sendo subutilizado. Tal subutilização deve-se não à falta de capacidade, e sim à pouca exploração dos dados que ele gera. Com os avanços da obtenção de dados e da analítica, estamos no limiar de uma nova era, em que o potencial máximo do TCPE, tanto para o prognóstico quanto para o diagnóstico, poderá ser revelado.

A eficiência ventilatória exemplifica uma área específica em que a total exploração dos dados do TCPE poderia revolucionar a maneira como tratamos a doença cardiopulmonar e cuidamos do paciente.^2^ Uma análise mais detalhada do TCPE revela que a inclinação VE/VCO_2_ (razão entre a ventilação minuto e a produção de dióxido de carbono) é uma ferramenta valiosa para navegar entre as complexidades de várias condições cardiopulmonares. Essa métrica, ao quantificar a eficiência ventilatória, serve como um importante indicador clínico para doenças como insuficiência cardíaca crônica (ICC), doença pulmonar obstrutiva crônica (DPOC), hipertensão arterial pulmonar, e doença pulmonar intersticial. Ainda, considerando a relação entre ventilação e demanda metabólica, a inclinação VE/VCO_2_ auxilia na classificação da gravidade da doença e na predição de risco de morbidade e de mortalidade. Assim, essa variável fornece informações importantes tanto sobre a eficiência do sistema respiratório quanto sobre a interação entre as funções pulmonar e cardíaca, levando ao direcionamento de estratégias personalizadas de manejo dos pacientes com condições cardiopulmonares.^3^

A inclinação VE/VCO_2_ exibe um comportamento variável entre as diferentes condições clínicas. Na DPOC, a obstrução das vias aéreas e as limitações mecânicas podem evitar a elevação esperada na inclinação com a progressão da doença.^4^ Por outro lado, na ICC, o valor da inclinação é geralmente mais alto devido à ineficiência metabólica e ao desequilíbrio entre a ventilação e a perfusão. Esses comportamentos contrastantes requerem uma interpretação cuidadosa da inclinação VE/VCO_2_ para assegurar a avaliação precisa da função cardiopulmonar e do estado de saúde do paciente, já que refletem mecanismos fisiopatológicos distintos de cada condição. É importante destacar que, embora tanto a DPOC como a ICC tenham um impacto sobre a eficiência ventilatória, seus mecanismos diferem-se significativamente. A DPOC exibe uma diminuição paradoxal na inclinação VE/VCO_2_ devido às limitações mecânicas e distúrbios de troca gasosa. Por outro lado, na ICC, o valor da inclinação é bem elevado, refletindo sua própria fisiopatologia.^5^

Orro et al.,^6^ em um artigo fascinante publicado nesta edição dos Arquivos Brasileiros de Cardiologia, apresentam uma nova abordagem para avaliar a eficiência ventilatória: o ηVE. Os autores destacam várias vantagens associadas a esse método:

## Melhor análise comparativa:

o método permite comparações mais diretas da eficiência ventilatória entre pacientes com ICC e pacientes com DPOC, acomodando as diferenças na fisiopatologia dessas condições.

## Útil na doença obstrutiva avançada:

ηVE é particularmente benéfico para avaliar pacientes com superposição de DPOC e ICC, em que métodos tradicionais como a inclinação VE/VCO_2_ podem ser menos eficaz dada à complexa interação entre as doenças.

## Supera limitações de métodos tradicionais:

Na DPOC, em que os mecanismos respiratórios e as doenças das vias aéreas afetam significativamente a eficiência ventilatória, a inclinação VE/CO_2_ pode levar a falsas interpretações. O ηVE oferece uma avaliação mais precisa nesses cenários.

O ηVE, conforme apresentado por Orro et al.,^6^ é uma ferramenta potencialmente mais refinada e precisa para avaliar a eficiência ventilatória no contexto das doenças cardiopulmonares. Isso é particularmente relevante quando métodos tradicionais podem ser inadequados pela complexidade inerente dessas condições. Os dados multifacetados obtidos pelo TCPE, incluindo variáveis bem estabelecidas, tais como a inclinação VE/VCO_2_ bem como o recentemente apresentado ηVE, parecem promissores na identificação de novos marcadores prognósticos.

Finalmente, a capacidade do TCPE em avaliar de maneira abrangente os diversos aspectos da saúde cardiopulmonar destaca seu valor significativo nos cenários clínicos. Ele transcende métricas convencionais como o consumo de oxigênio de pico – VO_2_ pico – ao fornecer *insights* mais profundos sobre a capacidade funcional e a progressão da doença dos pacientes.
