# Supplementary material for: A Eficiência Ventilatória Seria a Chave para Revelar o Potencial Máximo do Teste Cardiopulmonar de Exercício?
Source: Arq Bras Cardiol. 2024 May 17;121(4):e20240184. [Article in Portuguese] doi: 10.36660/abc.20240184 (PMC11164441; doi:10.36660/abc.20240184)
Supplement: Translation

# Translation: Ventilatory Efficiency: Is This the Key to Unlock the Full Potential of Cardiopulmonary Exercise Testing?

**DOI:** 10.36660/abc.20240184i

**Affiliations:** 1 Porto Alegre RS Brazil Grupo de Pesquisa em Cardiologia do Exercício (CardioEx) - Hospital de Clínicas de Porto Alegre, Porto Alegre, RS – Brazil; 2 Rio de Janeiro RJ Brazil LPH-Laboratório de Performance Humana, Rio de Janeiro, RJ – Brazil; 3 Rio de Janeiro RJ Brazil Universidade do Estado do Rio de Janeiro, Rio de Janeiro, RJ – Brazil; 4 Porto Alegre RS Brazil Programa de Pós-Graduação em Cardiologia e Ciências Cardiovasculares - Faculdade de Medicina, Universidade Federal do Rio Grande do Sul, Porto Alegre, RS – Brazil; 5 Porto Alegre RS Brazil Universidade Federal do Rio Grande do Sul - Departamento de Clínica Médica, Porto Alegre, RS – Brazil

**Keywords:** Ergoespirometry, Gas exchange, Markers

If Professor Jorge Pinto Ribeiro were still alive, he would undoubtedly be the one writing this Mini Editorial instead of us. Sadly, we lost him in 2013, but his memory and insightful words and articles live on. Professor Ribeiro was a passionate advocate for cardiopulmonary exercise testing (CPET). He authored numerous articles on the subject, including one with the thought-provoking title: "Beyond Peak Oxygen Uptake: New Prognostic Markers from Gas Exchange Exercise Tests in Chronic Heart Failure".^1^ It is an honor to mention that Professor Chiappa and Professor Stein were fortunate to collaborate with him on this piece.

So, what happened 18 years ago^1^ is directly related to what come next: although possessing a wealth of valuable data, the CPET remains underutilized. This underutilization stems not from a lack of capability, but rather from limited exploration of the data it generates. With advancements in data mining and analytics, we stand on the threshold of a new era where the CPET’s full potential, for both prognosis and diagnosis, can be unlocked.

Ventilation efficiency exemplifies a specific area where fully exploring CPET data could revolutionize the way we manage cardiopulmonary disease and deliver patient care.^2^ Delving deeper into the realm of CPET reveals the VE/VCO_2_ slope (minute ventilation to carbon dioxide production ratio) as a valuable tool for navigating the complexities of various cardiopulmonary conditions. This metric, quantifying ventilatory efficiency, serves as an important clinical indicator for diseases like chronic heart failure (CHF), chronic obstructive pulmonary disease (COPD), pulmonary arterial hypertension, and interstitial lung disease. In addition, considering the connection between ventilation and metabolic demand, the VE/VCO_2_ slope aids in classifying disease severity and predicting morbidity and mortality risks. Thus, this variable provides crucial insights into both respiratory system efficiency and the interplay between pulmonary and cardiac function, ultimately guiding personalized management strategies for patients with cardiopulmonary conditions.^3^

The VE/VCO_2_ slope exhibits changeable behavior across different clinical conditions. In COPD, airway obstruction and mechanical limitations can prevent the expected rise in the slope with disease progression.^4^ Conversely, CHF often presents with a higher slope due to metabolic inefficiencies and ventilation-perfusion mismatches. These contrasting behaviors require a nuanced interpretation of the VE/VCO_2_ slope to ensure accurate assessment of patient’s cardiopulmonary function and health status, as they reflect the distinct pathophysiological mechanisms underlying each condition. It is important to point out that, although both COPD and CHF impact ventilatory efficiency, their mechanisms differ significantly. COPD exhibits a paradoxical decrease in the VE/VCO_2_ slope due to mechanical limitations and gas exchange disruptions. Conversely, CHF presents with a distinctively higher slope, reflecting its unique pathophysiology.^5^

Orro et al.^6^ in a fascinating article published in this issue of *Arquivos Brasileiros de Cardiologia*, introduce a novel approach for assessing ventilatory efficiency: ηVE. The authors highlight several advantages associated with this method:

## Enhanced Comparative Analysis:

It allows for more direct comparisons of ventilatory efficiency between CHF and COPD patients, accommodating the pathophysiological differences between these conditions.

## Useful in Advanced Obstructive Disease:

ηVE is particularly beneficial for assessing patients with overlapping COPD and CHF, where traditional methods like the VE/VCO_2_ slope might be less effective due to the complex interplay of diseases.

## Overcoming Traditional Method Limitations:

In COPD, where respiratory mechanics and airway diseases significantly affect ventilatory efficiency, the VE/CO_2_ slope can be misleading. ηVE offers a more accurate assessment in such scenarios.

ηVE, as presented by Orro et al.,^6^ offers a potentially more refined and accurate tool for assessing ventilatory efficiency in the context of cardiopulmonary diseases. This is particularly relevant when traditional methods may prove inadequate due to the inherent complexities of these conditions. The multifaceted data obtained via CPET, including well-established variables like the VE/VCO_2_ slope and the recently introduced ηVE, hold promise for uncovering novel prognostic markers.

Finally, CPET’s ability to comprehensively evaluate diverse aspects of cardiopulmonary health underscores its significant value in clinical settings. It transcends conventional metrics like peak oxygen consumption - VO_2_ peak - by providing deeper insights into patients’ functional capacity and disease progression.
